# Quantitative comparison of food-based dietary guidelines: alignment with the Slovenian nutrition guidelines 2025 and Slovenian intake

**DOI:** 10.1007/s00394-026-04009-4

**Published:** 2026-06-02

**Authors:** Samo Kreft, Eva Tavčar, Anne Carolin Schäfer, Nataša Sivec, Zlatko Fras, Boštjan Jakše, Nataša Fidler Mis

**Affiliations:** 1 Sodobna Fitoterapija, 6215 Divača, Slovenia; 2 Etri Group, 1000 Ljubljana, Slovenia; 3 Faculty of Pharmacy, University of Ljubljana, 1000 Ljubljana, Slovenia; 4 German Nutrition Society, 53175 Bonn, Germany; 5 Nutritional Epidemiology, Department of Nutrition and Food Sciences, University of Bonn, 53115 Bonn, Germany; 6 Division of Medicine, Centre for Preventive Cardiology, University Medical Centre Ljubljana, 1000 Ljubljana, Slovenia; 7 Faculty of Medicine, University of Ljubljana, 1000 Ljubljana, Slovenia; 8 Independent Researcher, 4280 Kranjska Gora, Slovenia; 9 Independent Researcher, 1000 Ljubljana, Slovenia

**Keywords:** Food-based dietary guidelines, Dietary recommendations, Plant-forward diet, Sustainable diet, International comparison, Food groups, EAT–lancet, Nordic nutrition recommendations

## Abstract

**Purpose:**

Food-based dietary guidelines (FBDGs) are a key instrument for translating nutrition science into public health advice, yet direct comparison across guidelines is hindered by differences in food grouping, units, and formulation of recommendations. Slovenia has recently developed and scientifically completed the Slovenian Nutrition Guidelines 2025 (SNG2025), its updated FBDGs, which have been submitted for governmental adoption but are not yet officially adopted. The SNG2025 extend conventional FBDGs into a quantitative, plant-forward framework that integrates health and environmental sustainability. However, their quantitative position relative to contemporary international guidelines and nationally representative intake data has not yet been systematically evaluated.

**Methods:**

We compared 13 national FBDGs (Denmark, Finland, Sweden, Norway, Germany, Austria, Spain, France, Canada, United States, China, United Kingdom and Australia) and two international frameworks (Nordic Nutrition Recommendations and EAT–Lancet) with the SNG2025. Recommendations were extracted for major food groups and mathematically converted (standardised) to a common unit (g/day) using predefined conversion factors and explicitly stated assumptions.

**Results:**

The SNG2025 are largely consistent with international practice. Strong convergence was observed for fruits and vegetables, while recommendations for legumes, nuts, and whole grains closely aligned with EAT–Lancet targets and those from Spain and Austria. Slovenian upper limits for meat, dairy and eggs were comparable to those in several European and international guidelines; notably, some FBDGs suggest more stringent reductions than the SNG2025. Several guidelines allow fortified plant-based alternatives to fully replace animal-based dairy. Greater variability was observed for fish and seafood, reflecting regional dietary traditions. Significant heterogeneity in the formulation of recommendations (e.g., portion-based vs. weight-based) was identified.

**Conclusions:**

The SNG2025 are well aligned with contemporary international and national recommendations and align with widely accepted quantitative ranges for all major food groups. Differences between guidelines reflect cultural framing and quantification rather than conflicting nutritional principles. The SNG2025 demonstrate that contemporary dietary guidelines can integrate quantitative guidance, a plant-forward approach, and sustainability considerations while remaining aligned with international evidence. These findings support the use of SNG2025 as a reference framework for the development and evaluation of future dietary guidelines in comparable settings.

**Supplementary Information:**

The online version contains supplementary material available at 10.1007/s00394-026-04009-4.

## Introduction

Food-based dietary guidelines (FBDGs) are a central public health tool for translating nutritional science into practical recommendations for populations. In recent years, many countries have revised their guidelines to reflect advances in nutrition science, growing evidence on diet-related non-communicable diseases, and increasing awareness of environmental sustainability and food-system impacts [1, 2]. As a result, contemporary FBDGs differ substantially from versions published several decades ago, not only in the recommended quantities of specific food groups but also in their conceptual framing, such as the balance between nutrient-based and food-based approaches, the integration of sustainability considerations, and the resulting treatment of animal-source foods [1, 3].

Slovenia, as a small country, has faced constraints in independently developing all guidelines. Consequently, when preparing national guidelines, Slovenia has traditionally drawn extensively on international documents and on the experience of other countries, particularly those with well-established guideline-development processes. This pragmatic approach improves efficiency and scientific robustness but also raises an important question: to what extent are the quantitative recommendations adopted or adapted in Slovenia aligned with contemporary international and national guidelines elsewhere, and where do they meaningfully diverge?

Over the past several years, Slovenia has been actively preparing updated national food-based dietary guidelines. This process has been described in recent scientific and policy-oriented publications, which outline the use of national dietary intake data, evidence reviews, and alignment with international frameworks as core elements of guideline development [4–7]. These publications emphasise that Slovenian guidelines are intended to be evidence-based and culturally appropriate. At the same time, they highlight the importance of critically evaluating international recommendations rather than adopting them unmodified.

Several international analyses have demonstrated that a notable degree of heterogeneity exists among national dietary guidelines, reflecting cultural differences as well as variation in food preferences and food availability across countries, even when the guidelines are based on broadly similar scientific evidence [1, 8–13]. Differences are particularly pronounced for food groups such as meat, fish, dairy products, and fats, whereas recommendations for fruits and vegetables tend to be more consistent across countries. However, existing international comparisons have generally focused on broad descriptive differences between guidelines, while in this study we have systematically harmonised quantitative recommendations across food groups in a way that allows direct comparison with newly developed national guidelines and with observed national dietary intake. In particular, no such analysis has yet been conducted for the newly completed SNG2025. As a result, it remains unclear whether the Slovenian recommendations fall within the range of contemporary international practice, where they diverge, and how they relate to actual dietary intake in Slovenia.

In this context, a systematic and transparent comparison of SNG2025 with other contemporary national and international guidelines is essential. Such a comparison can clarify whether the SNG2025 are closely aligned with international practice, where they represent deliberate national choices, and where further refinement or clarification may be warranted. Moreover, quantitative standardisation of food-group recommendations enables downstream analyses, including comparisons with observed dietary intakes and modelling of health and environmental impacts.

The aim of this study is therefore to compare quantitative food-group recommendations from recently issued or updated dietary guidelines across multiple countries and international frameworks, including newly completed but unadopted SNG2025, and as a separate contextual component to place these recommendations in the context of actual dietary intakes in Slovenia. By systematically standardising recommendations across food groups and documenting all methodological assumptions, this study addresses the current lack of a transparent quantitative benchmark for interpreting the SNG2025 in an international and national context. The SNG2025 are scientifically finalised and have undergone full external peer review, but at the time of writing are awaiting formal governmental adoption and are therefore not yet legally binding.

## Methods

### Selection of dietary guidelines

We conducted a comparative analysis of the SNG2025 with other national and international FBDGs and reference diets that are currently in use, recently updated, or finalised but not yet formally published. Guidelines were eligible for inclusion if they: (i) provided food-based dietary guidance for the general adult population; (ii) were national guidelines, regional scientific frameworks, or internationally influential reference diets; (iii) contained sufficiently specific recommendations for the major food groups analysed in this study; and (iv) were available in an official or otherwise authoritative source document. The selection was guided by the aim of creating a representative benchmark for the SNG2025 in terms of geographical coverage and cultural diversity. Priority was given to FBDGs released or substantially revised within the last five years to reflect contemporary nutritional science; older guidelines were included only where they represent globally influential benchmarks. The final cohort encompassed international reference FBDGs (EAT–Lancet 1.0 and 2.0 [14, 15]), regional scientific frameworks (Nordic Nutrition Recommendations 2023 [16]), and national guidelines from Europe (Denmark [17], Finland [18], Sweden [19], Norway [20], Germany [21], Austria [22], Spain [23], France [24]), North America (Canada [25], the United States [26, 27]), Australia [28], and China [29]. The SNG2025 version used in this analysis corresponds to the final scientific version, incorporating feedback from national and international expert consultations. The completed SNG2025 were formally submitted to the Minister of Health of Slovenia on 8 October 2025 and are currently undergoing formal governmental approval. At the time of writing, they are not yet legally binding.

### Data extraction

For each FBDG, quantitative and qualitative recommendations were systematically extracted for predefined food groups, including vegetables, fruits, grains, potatoes and other starchy tubers, legumes, nuts and seeds, eggs, total meat (excluding fish and seafood), fish and seafood, milk and dairy products, and fats and oils. Given the well-recognised heterogeneity in food group classification across dietary guidelines, we applied a predefined harmonisation approach. Food categories with particularly inconsistent classifications and reporting across guidelines—such as beverages (including coffee), alcoholic beverages, sweets, and ultra-processed foods—were excluded from the analysis. For the remaining food groups, classifications were aligned to a common framework based on the Slovenian dietary guidelines, which are broadly consistent with widely used reference frameworks such as the EAT–Lancet reference diet. Where guidelines used different groupings (e.g., combining starchy vegetables and grains, or presenting fruits and vegetables jointly), recommendations were disaggregated based on the accompanying textual descriptions, where available. Similarly, observed differences in the classification of meat (e.g., red, white, and processed meat) were resolved by mapping reported recommendations to a common “total meat” category, while preserving distinctions where explicitly provided. In this harmonised category, “total meat” included all terrestrial animal meats reported in the guidelines, such as red meat, pork, beef, poultry (including chicken), and processed meat, but excluded fish and seafood, which were analysed separately. When guidelines reported only specific subcategories of meat, these were mapped to the common total meat category as far as possible, and the original distinctions were retained in the table notes where relevant.

This approach enabled consistent extraction and comparison of recommendations despite structural differences among guidelines. Where available, the verbatim wording of the recommendations was recorded and translated into English to ensure comparative consistency. When guidelines provided multiple formulations (e.g. frequency-based, portion-based, or narrative recommendations), all relevant information was captured and prioritised according to its normative status within the guideline (e.g., explicit quantitative limits over illustrative examples).

We also extracted qualitative recommendations regarding the diet, food preparation, and food choice, but not the explanation about the nutrients in the food or other information which does not give a direct recommendation to the consumer.

### Standardisation of quantities

To enable cross-guideline comparison, recommendations were mathematically converted (standardised) to **grams per day (g/day)** using food-group-specific conventions that reflect common practice and the nature of the food:

- **Grains, cereal products** and **legumes** were standardised as **dry weight**, using conversion factors explicitly stated in the guidelines where available; otherwise, a standardised conversion (e.g., 2:1 for cooked to dry weight) was applied and clearly documented.
- **Milk and dairy products** were standardised using **milk calcium equivalents** (MCE): 1 MCE (≈ 300 mg Ca) is provided by any one of the following: 250 mL of milk, 250 g of yoghurt, 250 mL of calcium-fortified plant drink, 250 g of calcium-fortified soy yoghurt, ≈ 50–75 g of soft cheese, and ≈ 27–42 g of hard/semihard cheese.

**Vegetables, potatoes, eggs, meat, fish, nuts and seeds, and fats and oils** were standardised as “as consumed” quantities. Here, “as consumed” refers to the edible quantity of food in the form in which it is usually eaten (e.g., cooked, prepared, or ready-to-eat), rather than its raw, uncooked, or dry-weight form.

When recommendations were expressed per week, monthly, or per serving, values were converted to daily equivalents. If portion sizes or equivalence factors were defined within the guideline document, these were used preferentially. No conversions were applied where guidelines provided only qualitative advice or energy-percentage (E%) targets without sufficient information to derive food-group quantities. No adjustment was made for differences in the energy reference levels underlying the guidelines (e.g., 2000 vs. 2500 kcal/day), because the purpose of this analysis was to compare recommendations as stated in the original documents, without introducing additional assumptions for energy-based rescaling across food groups. All specific conversions, portion-size interpretations, and any additional assumptions required for standardisation that were not explicitly defined in the original guidelines are reported directly in the *Assumptions/conversions* column of each table.

### Observed dietary intake data

Observed intake data for Slovenia were obtained from the representative national dietary survey **SI.Menu 2017/18** [30]. These data were not re-scaled or converted (beyond the units and dry/cooked legume conversion) and were included to contextualise guideline recommendations rather than to imply direct methodological equivalence with guideline targets. These data were included as a separate contextual component to illustrate the distance between current Slovenian intake and the levels implied by the guidelines, rather than as a direct like-for-like comparison with guideline recommendations. Observed intake data in SI.Menu were reported using a more detailed food classification than the broader food groups used in the dietary guidelines. For comparison, relevant SI.Menu subcategories were aggregated into the harmonised food groups used in this study. For example, the grains and cereals guideline group was aligned with SI.Menu categories: bread and bakery products and cereal and cereal products, including pasta, rice, and breakfast cereals.

### Transparency and reproducibility

All assumptions, conversions, and methodological decisions are explicitly documented in the tables accompanying the results. Where multiple guideline documents or versions were consulted for a single country or framework, primary sources are listed separately from supplementary guideline-linked documents. This approach was adopted to ensure **transparency, reproducibility, and traceability** of the standardised values and to allow readers to critically assess the robustness of cross-guideline comparisons.

## Results

The analysis included 13 FBDGs —from Denmark, Finland, Sweden, Norway, Germany, Austria, Spain, France, Canada, the United States, China, the United Kingdom and Australia—as well as 2 international FBDGs, the Nordic Nutrition Recommendations (NNR 2023) and the EAT–Lancet reference diets (2019 and 2025 versions). These national and international guidelines were systematically compared with the newly developed SNG2025 and with a nationally representative intake (Si. Menu 2017/18) in Slovenia.

Table 1 provides an overview of all included FBDGs, including their publication year, description and primary sources. Subsequent Tables 2, 3, 4, 5, 6, 7, 8, 9, 10, 11, and 12 present **food-group-specific comparisons**, detailing for each guideline the **verbatim quantitative wording** as stated in the original document, and any **qualitative or additional guidance** related to food quality, preparation, or substitution. To ensure transparency and reproducibility, **explicit assumptions and conversion rules** applied during standardisation are reported alongside the standardised quantitative values in each table.

**Table 1:** Overview of guidelines included in this comparison

Short guideline label	Country/region & year	Description	Citation	Primary URL
SNG2025	Slovenia, 2025	SNG2025, developed and submitted to the Minister of Health of Slovenia (October 2025); not yet formally adopted	Fidler Mis N, Jakše B, Kreft S, Vovk A, Fras Z. Three-Tier Plate, Triple Win: Health, Sustainability, and Equity in the Slovenian Nutrition Guidelines 2025. Foods. 2026;15(4):656. 10.3390/foods15040656.1	10.3390/foods15040656
SI nat. repr. Intake	Slovenia, 2017/18 survey (paper 2022)	Slovenian national dietary survey; scientific article nationally representative intake data	Gregorič M, Hristov H, Blaznik U, Koroušić Seljak B, Delfar N, Pravst I. Dietary Intakes of Slovenian Adults and Elderly: Design and Results of the National Dietary Study SI.Menu 2017/18. *Nutrients.* 2022;14(17):3618. 10.3390/nu14173618	https://www.mdpi.com/2072-6643/14/17/3618
EAT–Lancet 2.0	Global, 2025	Scientific commission report; journal article (technical); 2400 kcal per day	Rockström J, et al. The EAT–Lancet Commission on healthy, sustainable, and just food systems. *Lancet.* 2025;406(10512):1625–1700. 10.1016/S0140-6736(25)01201-2	https://www.thelancet.com/journals/lancet/article/PIIS0140-6736(25)01201-2/fulltext
EAT–Lancet	Global, 2019	Scientific commission; journal article (reference diet with ranges); 2500 kcal per day	Willett W, et al. Food in the Anthropocene: the EAT–Lancet Commission on healthy diets from sustainable food systems. *Lancet.* 2019;393(10170):447–492. 10.1016/S0140-6736(18)31788-4	https://www.thelancet.com/journals/lancet/article/PIIS0140-6736(18)31788-4/fulltext
NNR 2023	Nordic & Baltic region, 2023	Technical scientific basis for national guidelines; report	Nordic Council of Ministers. *Nordic Nutrition Recommendations 2023 (NNR2023).* Copenhagen: Nordisk Ministerråd; 2023	https://pub.norden.org/nord2023-003/nord2023-003.pdf
DK	Denmark, 2021	National public guidelines; climate-health framing; PDF guide	Danish Veterinary and Food Administration. *The Official Dietary Guidelines – Good for Health and Climate.* Denmark; 2021	https://foedevarestyrelsen.dk/Media/638194807769097944/Danish_Official_Dietary_Guidelines_Good_for_Health_and_climate_2021_PRINT_ENG__webtil.pdf
FI	Finland, 2024	National recommendations; technical/public-facing PDF	Finnish National Nutrition Council (under Finnish Food Authority). *Sustainable Health from Food: Finnish Nutrition Recommendations 2024.* Finland; 2024	https://www.ruokavirasto.fi/globalassets/teemat/terveytta-edistava-ruokavalio/ravitsemus--ja-ruokasuositukset/sustainable-health-from-food_web.pdf
SE	Sweden, 2025	National public guidelines; updated with sustainability	Swedish National Food Agency (Livsmedelsverket). Dietary guidelines for adults. Sweden; 2025	https://www.livsmedelsverket.se/en/food-habits-health-and-environment/dietary-guidelines/
NO	Norway, 2024 (web update)	National public guidance portal (patient/public oriented)	Helsenorge. Kostrådene (dietary advice) (web). Norway; updated 2024	https://www.helsenorge.no/kosthold-og-ernaring/kostradene/
DE	Germany, 2024	National FBDG; web summary (public + methodological notes)	German Nutrition Society (DGE). Food-Based Dietary Guidelines for Germany (DGE recommendations “Eat and drink well”). Germany; 2024	https://www.dge.de/english/fbdg/
	Germany, 2025	Peer-reviewed methods paper supporting DGE 2024 FBDG	Schäfer AC, Boeing H, Gazan R, Conrad J, Gedrich K, Breidenassel C, et al. A methodological framework for deriving the German food-based dietary guidelines 2024: Food groups, nutrient goals, and objective functions. *PLOS ONE.* 2025;20(3) 10.1371/journal.pone.0313347	10.1371/journal.pone.0313347
AT	Austria, 2024/2025 (new recommendations released)	National recommendations; web hub + official PDF(s)	AGES (Austrian Agency for Health and Food Safety). Österreichische Ernährungsempfehlungen (new recommendations) (web). Austria; updated 2024/2025	https://www.ages.at/mensch/ernaehrung-lebensmittel/ernaehrungsempfehlungen/oesterreichische-ernaehrungsempfehlungen
ES	Spain, 2022	National-level healthy & sustainable dietary recommendations; PDF	AESAN (Agencia Española de Seguridad Alimentaria y Nutrición). *Recomendaciones dietéticas saludables y sostenibles.* Spain; 2022	https://www.aesan.gob.es/AECOSAN/docs/documentos/nutricion/RECOMENDACIONES_DIETETICAS.pdf
	Spain, 2025/26	Professional society guidelines; pyramid + detailed document	Sociedad Española de Nutrición Comunitaria (SENC). *Guías Alimentarias SENC 2025/26* (document). Spain; 2025	https://www.renc.es/imagenes/auxiliar/files/RENC-D-25-0001._Final.pdf
FR	France, 2019	Technical report (“state of knowledge” + quantified adult recs)	Santé publique France. *Recommendations concerning diet, physical activity and sedentary behaviour for adults.* France; 2019	https://www.santepubliquefrance.fr/content/download/515446/3807453?version=1
CN	China, 2022	National dietary guidelines; public + technical ecosystem	Chinese Nutrition Society (or national guideline consortium). *Dietary Guidelines for Chinese Residents (2022).* China; 2022	https://en.chinacdc.cn/health_topics/nutrition_health/202206/t20220616_259702.html Consulted also original Chinese, since the “official” English translation is partly ambiguous: https://wjw.hubei.gov.cn/bmdt/jkhb/spyy/202205/t20220519_4133461.shtml
US	USA, 2020–2025	Official federal dietary guidelines; report + policy basis (at the 2,000-cal)	U.S. Department of Agriculture & U.S. Department of Health and Human Services. *Dietary Guidelines for Americans, 2020–2025.* 9th ed. Washington, DC; 2020	https://www.dietaryguidelines.gov/
	USA, 2025–2030	Published after the end of data extraction for this article	U.S. Department of Agriculture & U.S. Department of Health and Human Services. Dietary Guidelines for Americans, 2025–2030: Work Under Way. USA; 2025	https://www.dietaryguidelines.gov/work-under-way
UK	United Kingdom, 2016 (updated 2024)	National public-facing food guide. F: 2000 kcal M: 2500 kcal	Public Health England. *The Eatwell Guide.* United Kingdom; 2016	https://www.gov.uk/government/publications/the-eatwell-guide
CA	Canada, 2019	National guidelines; report (food guide + principles) contain no quantitative information and are not included in subsequent tables	Health Canada. *Canada’s Dietary Guidelines.* Ottawa, Canada; 2019	https://food-guide.canada.ca/en/guidelines/
AU	Australia, 2013	National guidelines; current edition + revision process, (currently under review)	National Health and Medical Research Council (NHMRC). *Australian Dietary Guidelines.* Canberra, Australia; 2013	https://www.eatforhealth.gov.au/guidelines

^1^As SNG2025 has not yet been formally adopted, its scientific basis, as described in peer-reviewed publications, was used for comparison

**Table 2:** Quantitative and qualitative recommendations for grains/cereals across dietary guidelines, including verbatim recommendations, qualitative notes, assumptions/conversions, and standardised intake values expressed as **g/day (dry weight)**

Guideline	Verbatim quantitative recommendation (English translation)	Qualitative / extra notes (non-quantitative)	Assumptions / conversions	Standardised food group intake recommendation (g/day, dry weight)
SNG2025	We recommend at least six portions of grains per day (about 230 g raw or 600 g cooked). One portion = two slices of bread or 150 g cooked pasta/porridge/rice	Half should be whole grain, preferably more		≥ 230
SI nat. repr. intake	The study reports separately on bread and cereals	–	Sum of bread and cereal	Adults: ♂ 307.2, ♀ 239.2 Elderly: ♂ 274.1, ♀ 231.3
EAT–Lancet 2.0	Per capita recommended intake (g/day [range]): 210 g (20–50% of daily energy intake)			≈210
EAT–Lancet	Whole grains: 232 g/day (range 0–464 g/day)	Whole grains defined as staple		232 (0–464)
NNR 2023	It is recommended to consume at least 90 g/day (dry weight) of whole grains, with likely further benefits of higher intakes	Explicitly whole grains; refined grains may have a role at high energy needs; rice noted as a higher-impact exception		≥ 90
DK	Eat 75 g of whole grain a day or more	Choose wholegrain		≥ 75
FI	Minimum recommendation for whole grains: 90 g per day (dry weight)	Fibre content of soft breads should be at least 6%		≥ 90
SE	We recommend eating at least 90 g of whole grains per day, preferably more	Whole grains preferred; refined grains limited		≥ 90
NO	recommended that you eat at least 90 g of wholegrain daily	Encourages wholegrain; vary sources		≥ 90
DE	NA	Favour whole-grain foods		NA
AT	Eat 4 portions of grains or potatoes daily One portion of bread is roughly the size of your palm. One portion of pasta, or rice (cooked), is roughly the size of two fists	Choose from a variety of grains and opt for whole-grain varieties	Portion size 70 g. Assuming out of 4 portions cereals could be between 0 and 4 and the rest is potato	0–280
ES	Consume 3–6 servings a day, more servings if you live an active life with a high caloric expenditure and no more than 4 servings if you need to reduce caloric intake. Portion: 40–60 g or bread or 60–80 g of dry pasta or rice	Choose whole grain cereals such as wholegrain rice, bread, or pasta (preferably 100%), minimise refined	Calculation of interval using extreme values of 3 serving × 40 g and 6 servings × 80 g	120–480
FR	To be consumed daily	Wholegrain/unrefined instead of refined products		NA
CN	Consume 200–300 g of grains daily, including 50–150 g of whole grains and legumes			200–300
US	Whole Grains (ounce eq/day) ≥ 3 Refined Grains (ounce eq/day) < 3	Choose 100% whole-grain foods for at least half of all grains consumed	1 oz-eq = 28.35 g	Total Grains ≈170 Whole Grains ≥ 85
UK	Eat plenty of starchy carbohydrates;	Choose wholegrain		NA
AU	6 serves per day (serve is 40 g bread, 30 g muesli, 75–120 g cooked pasta)		6 × 40 = 240	≈240

**Table 3:** Quantitative and qualitative recommendations for potatoes/starchy tubers across dietary guidelines, including verbatim recommendations, qualitative notes, assumptions/conversions, and standardised intake values expressed as g/day (as consumed)

Guideline	Verbatim quantitative recommendation (English translation)	Qualitative / extra notes (non-quantitative)	Assumptions / conversions	Standardised food group intake recommendation (g/day, as consumed)
SNG2025	One portion per day (approximately 200 g) of potatoes or other tubers	Prefer boiled/baked; limit fried potatoes;		≈200
SI nat. repr. intake	Observed mean intake of potatoes (g/day)			Adults: ♂ 99.0, ♀ 75.7 Elderly: ♂ 99.0, ♀ 87.8
EAT–Lancet 2.0	Tubers or starchy roots: 50 g/day (range 0–100 g/day)			≈50 (0–100)
EAT–Lancet	Tubers or starchy vegetables (potatoes and cassava): 50 g/day			≈50 (0–100)
NNR 2023	Potatoes can be part of a diet. Should be included as a significant part of the regular dietary pattern	Prefer boiled or baked potatoes; limit fried products		NA
DK	Include potatoes several times a week. About 100 g/day of potatoes is adequate			≈100
FI	Potatoes are a recommended part of a healthy and environmentally friendly diet	High-fat and salty potato dishes should be avoided		NA
SE	Potatoes are also not included in the 500 g/day amount. It is certainly good food, but if a large portion of 500 g/day of vegetables consists of potatoes, the variety becomes too limited			NA
NO	Potatoes are part of a healthy and varied diet but are not included in the recommended intake for fruits, berries, and vegetables			NA
DE	NA			NA
AT	Eat 4 portions of grains or potatoes daily. One portion of potatoes is roughly the size of two fists		Portion size 150–200 g. Assumed it can be only potato or only grains	0–800
ES	Moderate the intake of potatoes and other tubers	Better to consume cooked or steamed potatoes		NA
FR	Bread, pasta, rice, flour, potatoes: 1 portion per meal	Potatoes are grouped with starchy foods; quantity depends on energy needs	Assumed 4 meals/day; portion size 150–200 g. Range reflects the extreme allocation within the combined ‘grains or potatoes’ instruction	0–800
CN	50–100 g/day of tubers			50–100
US	Starchy vegetables (cup eq/wk)		1 cup equivalent of cooked potatoes is 150–210 g	≈ 107–150
UK	Eat plenty of starchy carbohydrates	keep the skins on potatoes		NA
AU	Potatoes included in vegetables	Fried potatoes discouraged		NA

**Table 4:** Quantitative and qualitative recommendations for legumes/pulses across dietary guidelines with values standardised to g/day (dry weight) where possible

Guideline	Verbatim quantitative recommendation (English translation)	Qualitative/extra notes (non-quantitative)	Assumptions/conversions	Standardised food group intake recommendation (dry g/day)
SNG2025	Daily, at least one portion of cooked legumes (100 g/day, i.e., 50 g/day dry weight) and an additional 70 g/day cooked soybeans (25 g/day dry weight) or soy products	Soak before cooking; increase gradually; spices; sufficient fluids; sprouted/fermented forms may be easier to digest		≥ 75
SI nat. repr. intake	Legumes and legume-based products… Adults: 11.6 g/day (women), 14.6 g/day (men); Elderly: 17.4 g/day (women), 21.3 g/day (men)		Factor 2 for cooked → dry conversion. Note that this category may include items (e.g., tofu, hummus) where cooked → dry ≠ 2	Adults: ♂ 7.3, ♀ 5.8, Elderly: ♂ 10.7, ♀8.7
EAT–Lancet 2.0	Legumes: 75 g/day (0–150 g/day)			≈75 *(0–150)*
EAT–Lancet	Dry beans, lentils, and peas: 50 g/day (0–100) Soy foods: 25 g/day (0–50 g/day)			75 *(0–150)*
NNR 2023	Pulses should be included as a significant part in the regular dietary pattern			NA
DK	About 100 g/day is adequate	Eat more legumes, such as brown, white and black beans, kidney beans, lentils and chickpeas	Factor 2 for cooked → dry conversion	≈ 50
FI	A daily intake target of 50 to 100 g/day (cooked) is recommended	Emphasises gradual increase and variety	Factor 2 for cooked → dry conversion	≈ 25–50
SE	Eat beans, peas and lentils often – preferably every day			NA
NO	Use beans, lentils and peas for dinner or as a side dish		Not clear if it is meant for every dinner	NA
DE	Eat legumes at least once a week		Portion size + conversion factor taken from the technical/auxiliary DGE documentation [31].: dry = 125/1.8 = 69.4 g/week → 9.9 g/day dry	≥ 9.9
AT	Legumes at least 3 × /week (with meat/fish) or 4 × /week (without meat/fish); one serving ~ 125 g cooked. One serving of tofu or tempeh is around 80 g	Distinguishes dietary patterns	Assumed cooked-to-dry conversion factor 2:125 cooked → 62.5 dry; 3–4/wk → 26.8–35.7 g/day	≥ 26.8 *(omnivorous)* ≥ 35.7 *(vegetarian)*
ES	At least 4 servings a week until you get to consume them daily. Portion: 50–60 g dry or about 170 g already prepared	Soak the legumes. If canned, choose low-salt varieties	min: 4 × 50/7 = 28.6 max: 1 × 60 = 60	28.6–60
FR	Legumes at least twice per week (lentils, dried beans, chickpeas, etc.)	They can replace meat and poultry	Assumed portion size 50 g (dry) → 100 g/week → 14 g/day	≥ 14
CN	Consume 200–300 g/day, including 50–150 g /day of whole grains and legumes		Combined category (whole grains + legumes) → legumes-only g/day not extractable	NA
US	Beans, peas, lentils: 1.5 cup equivalents per week	Soy product grouped with nuts and seeds	1 cup equivalent (cooked) ≈ 172 g; 1.5 × 172 g / 7 = 36.9 g/day → 19 g dry (factor 2)	≈19
UK	Eat more beans, pulses. No numeric target			NA
AU	Legumes included within “lean meats and poultry, fish, eggs, tofu, nuts and seeds, and legumes/beans”: 3 portions per day			NA

**Table 5:** Quantitative and qualitative recommendations for fruits across dietary guidelines, expressed as g/day (as consumed)

Guideline	Verbatim quantitative recommendation (English translation)	Qualitative / extra notes (non-quantitative)	Assumptions / conversions	Standardised food group intake recommendation (g/day, as consumed)
SNG2025	Consume at least 200 g/day. Preferably between 100 and 300 g/day	Prefer whole fruit; limit fruit juice; emphasise variety/seasonality		≥ 200
SI nat. repr. intake	“Fruit (fresh and preserved/canned), mean intake (g/day) …”	Observed intake category includes fresh + preserved/canned fruit (survey classification)		Adults: ♂ 162.2, ♀ 226.0 Elderly: ♂ 210.7, ♀ 267.8
EAT–Lancet 2.0	200 g/day (range 100–300 g/day)			≈200 (100–300)
EAT–Lancet	Fruits: 200 g/day (range 100–300 g/day)			200 (100–300)
NNR 2023	Consume 500–800 g/day or more of vegetables, fruits and berries in total	includes berries	fruit assumed 40%: ≥ 0.4 × 500 = ≥ 200 g/day	≥ 200 (or > 320)
DK	Eat 600 g of vegetables and fruits per day – that is, "6 a day." At least half should be vegetables	A small glass of juice (100 ml) can count as 1 of your ‘6 a day’, but only as 1	fruit assumed 40%: ≥ 240 g/day	≥ 240
FI	Eat at least 500–800 g/day of vegetables, fruit and berries … about ½ should be berries and fruit	Frozen are good alternatives, Sweetened products are not recommended	Guideline states ≈50% berries + fruit → ≥ 0.5 × 500 = ≥ 250 g/day (and if 800 → 400)	≥ 250 (≥ 400)
SE	Eat lots of vegetables, fruit and berries, at least 500 g/day and preferably more		fruit assumed 40%: ≥ 0.4 × 500 = ≥ 200 g/day	≥ 200
NO	Eat at least five and preferably eight daily servings of fruits, berries and vegetables; one serving is 100 g; roughly half fruit/berries and half vegetables	Up to 1 dl of juice can be counted as 1 serving if the juice is made from 100% fruit, berries or vegetables. Juice should not be drunk outside of meals	≥ 5–8 servings → ≥ 500 – ≥ 800 g, half are fruits and berries → ≥ 250 – ≥ 400 g,	≥ 250 (≥ 400)
DE	Enjoy at least five portions of fruit and vegetables daily	Fruit and vegetables combined in “5-a-day” message	Portion size 100 g: total fruits + vegetables ≥ 500 g/day; fruit assumed 40% → ≥ 200 g/day	≥ 200
AT	Eat 5 portions/day, ideally 3 portions of vegetables and 2 portions of fruit	Choose colorful and varied	Portion size assumed 100 g	≈200
ES	2–3 servings of fruit per day	Consuming fruit juices is not a substitute for whole fruits	Fruit servings 2/day; fruit serving size not fixed here → assumed 120 g/serving (conservative) → 240 g/day (and if 3 servings → 360 g/day)	240–360
FR	At least 5 portions of fruits and vegetables per day (80 –100 g/day)	No more than a glass of fruit juice per day	total fruit + veg ≥ 400 g/day; fruit assumed 40% → ≥ 160 g/day	≥ 200
CN	A daily intake of 200–350 g/day of fresh fruits is recommended	they cannot be replaced by processed fruit juice		200–350
US	Fruit quantified within dietary patterns, e.g., ~ 2 cup-equivalents/day in a 2,000-kcal pattern	Whole fruits include fresh, canned, frozen, and dried. At least ½ should come from whole fruit, rather than 100% juice	assumption 1 cup-equivalent fruit ≈ 150 g as eaten → 2 × 150 ≈ 300 g/day	≈300
UK	Eat at least 5 portions of a variety of fruit and vegetables every day		Portion size assumed 100 g/portion: total fruit + vegetables ≥ 500 g/day; fruit assumed to be 40% → ≥ 200 g/day	≥ 200
AU	Minimum recommended number of serves of fruit per day: 2 (serving size is 150 g)		≥ 2 × 150 g = ≥ 300 g/day	≥ 300

Where guidelines specify a combined fruit + vegetable target without a split, it is assumed that the fruit share constitutes 40% of the total

**Table 6:** Quantitative and qualitative recommendations for vegetables across dietary guidelines. Where guidelines specify a combined fruit + vegetable target without a split, an assumption was made, that vegetables share is 60% of the total

Guideline	Verbatim quantitative recommendation (English translation)	Qualitative/extra notes (non-quantitative)	Assumptions/conversions	Standardised food group intake recommendation (g/day, as consumed)
SNG2025	Consume at least 300 g/day of vegetables	especially cruciferous, dark green leafy and colorful vegetables		≥ 300
SI nat.repr. intake	Vegetables (fresh and preserved/canned), mean intake (g/day)	Observed intake category includes fresh + preserved/canned vegetables (survey classification)		Adults: ♂ 163.0, ♀ 158.3 Elderly: ♂ 160.1, ♀ 163.2
EAT–Lancet 2.0	300 g/day (200–600 g/day)			≈300 *(200–600)*
EAT–Lancet	All vegetables 300 g/day (200–600 g/day). Dark green vegetables: 100 g/day Red and orange vegetables: 100 g/day Other vegetables: 100 g/day			≈300 *(200–600)*
NNR 2023	Consume 500–800 g/day or more of vegetables, fruits and berries in total	Potatoes/tubers are treated separately from vegetables, fruits and berries	no split stated → vegetables assumed 60%: ≥ 0.6 × 500 = ≥ 300 g/day	≥ 300 *(or* ≥ *480)*
DK	Eat at least 600 g of vegetables and fruit per day … at least half should be vegetables	Potatoes not counted within the 600 g fruit + vegetables; eat different types: high fibre, dark green, red, orange	Explicit split in guideline: vegetables ≥ 50% of total → ≥ 300 g/day	≥ 300
FI	Eat at least 500–800 g/day of vegetables, fruit and berries … about half should be vegetables (incl. root vegetables)	Root vegetables are explicitly included on the vegetable side	Guideline states ≈50% vegetables → 0.5 × 500 = 250 g/day; 0.5 × 800 = 400 g/day; “at least” retained as ≥	≥ 250 (can be > 400)
SE	Eat at least 500 g of vegetables and fruit every day	Rough vegetables, such as root vegetables, cabbage, cauliflower, broccoli and onions, are especially good	vegetables assumed 60%: ≥ 0.6 × 500 = ≥ 300 g/day	≥ 300
NO	Eat at least five and preferably eight daily servings of fruits, berries and vegetables; one serving is 100 g; roughly half fruit/berries and half vegetables	Limit intake of processed vegetable products with added salt and fat	≥ 5–8 servings → ≥ 500 – ≥ 800 g/day, half are fruits and berries → ≥ 250 – ≥ 400 g/day	≥ 250 (≥ 400)
DE	Enjoy at least five portions of fruit and vegetables daily, …	Potatoes are presented separately from vegetables in the DGE food circle (i.e., not counted as vegetables)	Portion size 100 g: total fruit + veg ≥ 500 g/day; vegetables 60% → ≥ 300 g/day	≥ 300
AT	Eat 5 portions/day, ideally 3 portions of vegetables and 2 portions of fruit	Eat some vegetables raw (e.g., in salads). Choose colorful and varied	Portion size assumed 100 g	≈300
ES	At least 3 servings of vegetables. Vegetable serving: 150–200 g	Increase consumption of cruciferous vegetables (sprouts, cabbages, and radishes), dark green leafy vegetables (spinach, chard, etc.)	Vegetables ≥ 3 servings/day; using the stated lower bound 150 g/serving → ≥ 450 g/day (upper if 200 g/serving: ≥ 600 g/day)	≥ 450—*600*
FR	At least 5 portions of fruits and vegetables per day (80–100 g)	The recommendation is to increase your consumption regardless of the initial consumption level	Total fruit + veg ≥ 400 g/day; vegetables assumed 60% → ≥ 240 g/day	≥ 240 (or ≥ 300)
CN	Recommended daily intake of vegetables is 300–500 g	≥ 50% dark-coloured vegetables		300–500
US	Dark-Green veg. (cup eq/wk): 1 ½ Red and Orange veg. (cup eq/wk): 5 ½ Other veg. (cup eq/wk): 4	Subgroups are emphasised (dark green, red/orange, legumes, etc.)	Assumption 1 cup-equivalent vegetables ≈ 150 g as eaten → 11 × 150/7 ≈ 236 g/day	≈236
UK	Eat at least 5 portions of a variety of fruit and vegetables every day	Potatoes and other starchy staples do not count toward “5-a-day”	Portion of 100 g: vegetables assumed to constitute 60%	≥ 300
AU	Minimum recommended number of serves of vegetables per day: 6 for man, 5 for woman (1 serving = 75 g)		Standard serving ≈75 g → vegetables ≥ 5 × 75 = ≥ 375 g/day	≥ 375

**Table 7:** Quantitative and qualitative recommendations for nuts & seeds across dietary guidelines, expressed as g/day (as consumed)

Guideline	Verbatim quantitative recommendation (English translation)	Qualitative/extra notes (non-quantitative)	Assumptions/conversions	Standardised food group intake recommendation (g/day, as consumed)
SNG2025	It is recommended to consume at least 30 g of nuts and seeds daily (a small handful), either as a snack or added to meals	Includes tree nuts (e.g., hazelnuts, walnuts, almonds, pistachios) and seeds (pumpkin, flax, sesame, sunflower, chia)		≥ 30
SI nat. repr. intake	Category reported as “fresh and processed nuts and seeds”			Adults: ♂ 7.7; ♀ 9.5 Elderly: ♂ 4.5; ♀ 6.8
EAT–Lancet 2.0	Tree nuts and peanuts 50 g/day (0–75 g/day)			≈50 (0–75)
EAT–Lancet	Peanuts: 25 g/day (0–75 g /day) Tree nuts: 25 g/day			≈50
NNR 2023	Consume 20–30 g nuts per day. Recommended to include seeds, evidence for a quantity is not available	Choosing unsalted/ minimally processed options		20–30 (nuts + seeds)
DK	Eat about 30 g of nuts a day About 1–2 tablespoons of seeds a day is adequate	Choose unsalted nuts or nuts with a maximum of 0.8 g of salt per 100 g	Assumed: 1 tablespoon seeds ≈10 g; sumarised seeds and nuts	≈40–50
FI	20 to 30 g of unflavoured nuts daily, depending on the type of nuts, is recommended. Also include a variety of different seeds		The seeds are not quantified	nuts: 20–30 seeds: NA
SE	Eat two to three tablespoons of unsalted nuts a day. Seeds are also good for your health		assumed tablespoon size = 10 g	nuts: 20–30 seeds: NA
NO	Recommended to eat 20 to 30 g of nuts every day. It is also recommended to include seeds in your diet	unsalted		nuts: 20–30 seeds: NA
DE	Eat a small handful of nuts daily	Prefer unsalted	assumed portion = 25 g	25
AT	Oils, fats, nuts & seeds — daily: 2 portions. A portion seeds/nuts ≈2 tbsp.		Assuming a portion is 20 g, and both recommended portions consist of seeds/nuts, the maximum intake would be ≈40 g/day (since portions may be oils and fats instead)	≤ 40
ES	It is recommended to consume 3 or more servings a week until you reach a daily serving intake. Portion: 20–30 g	Choose unsalted nuts without added fats or sugars”; energy density noted balance with other energy sources	Converted from weekly minimum: 3 servings/week × 20 g ÷ 7 = 8.5 g/day (minimum). Upper end reflects “daily” (30 g/day)	8.5–30
FR	A small handful per day	Unsalted	Assumed 1 portion ≈20 g	≈20
CN	Appropriate amount of nuts should be consumed too			NA
US	Healthy U.S.-Style Pattern (2,000 kcal): nuts, seeds, soy products: 5 oz-equivalents per week	Unsalted/unsweetened options implied via overall pattern guidance	1 oz-eq = 28.35 g 5 × 28.35 /7 = 20 g/day This group includes soy products	≈20
UK				NA
AU	Nuts and seeds are included within the group 'lean meats and poultry, fish, eggs, tofu, nuts and seeds, and legumes/beans': 3 portions per day			21

**Table 8:** Quantitative and qualitative recommendations for fish & seafood across dietary guidelines expressed as g/day (as consumed)

Guideline	Verbatim quantitative recommendation (English translation)	Qualitative / extra notes (non-quantitative)	Assumptions / conversions	Standardised food group intake recommendation (g/day, fish + seafood “as consumed”)
SNG2025	It is recommended to consume ~ 200 g per week (1–2 portions), (range: 0–450 g/week)	Prefer oily fish; sustainability/contaminant considerations	Converted from weekly range (÷ 7)	≈29 (range 0–64)
SI nat. repr. intake	Observed mean intake of “fish and fish products” (SI.Menu 2017/18 analysis)			Adults: ♂ 26.5; ♀ 18.2 Elderly: ♂ 19.8; ♀ 21.2
EAT–Lancet 2.0	Fish and shellfish: 30 g/day (0–100 g/day)			≈ 30 (0–100)
EAT–Lancet	Fish: 28 g/day (range 0–100 g/day			≈ 28 (0–100)
NNR 2023	300–450 g fish/week (ready-to-eat weight), of which ≥ 200 g/week should be fatty fish	Emphasises sustainability; include fatty + lean varieties	Weekly → daily (÷ 7)	43–64
DK	Eat 350 g of fish per week, of which 200 g is oily fish	It is important to eat different types of fish	Weekly → daily (÷ 7)	50
FI	Fish and fish products 300–450 g/week (cooked, edible part), of which ≥ 200 g/week should be fatty fish	Prefer sustainable stocks; vary species	Weekly → daily (÷ 7)	43–64
SE	Eat fish and shellfish 2 to 3 times a week	vary fatty/lean	Assumed 150 g per fish meal (2–3 × /week ⇒ 300–450 g/week)	≈43–64
NO	Eat fish and seafood two to three times a week… recommended 300 to 450 g of fish every week… at least 200 g should be fatty fish	Includes fish as spread; variety (fatty + lean)	Weekly → daily (÷ 7)	43–64
DE	Eat fish once to twice a week	Highlights fatty fish (omega-3) and saltwater fish (iodine)	Assumed 150 g per fish meal. Weekly → daily (÷ 7)	≈21.4–42.8
AT	Eat 1 portion of fish per week (a portion = a palm-sized, finger-thick piece)	If you avoid saltwater fish, consume an additional 1 tablespoon of rapeseed oil per day	Assumed 150 g per fish portion. Weekly → daily (÷ 7)	≈21.4
ES	Consume at least 3 servings a week, Portion: 125–150 g	Prioritising oily fish (blue: sardines, anchovies, mackerel, scad, etc.; white: haddock, whiting, cod, etc.; seafood: mussels, etc.)	Weekly → daily (÷ 7)	≥ 53.6–64.3
FR	Consume fish twice a week, including fatty fish	Vary the species and the supply locations (especially in case of high consumption) to limit exposure to contaminant	Assumed 150 g per fish portion. Weekly → daily (÷ 7)	≈42.8
CN	Recommended weekly intakes: fish 300–500 g (or twice a week)	Fish prioritised within animal foods; also frames total animal-food intake	Weekly → daily (÷ 7)	43–71
US	Seafood (ounce eq/wk): 8	Encourages seafood choices; mercury guidance exists for specific groups	8 oz/week ≈ 227 g/week → ≥ 32 g/day	≥ 32
UK	Aim for at least 2 portions (2 × 140 g) of fish every week, 1 of which should be oily	Oily fish at least once/week; sustainability/healthy choices context	280 g/week → ≥ 40 g/day	≥ 40
AU	For adult Australians who consume foods from animal sources, around 2 servings of fish per week are recommended		2 serves → 200 g/week → ≈29 g/day	≈29

**Table 9:** Quantitative and qualitative recommendations for milk & dairy across dietary guidelines

Guideline	Verbatim quantitative recommendation (English translation)	Qualitative / extra notes (non-quantitative)	Assumptions / conversions	Standardised food group intake recommendation (g/day, milk equivalents)
SNG2025	A reasonable daily intake may include approximately: 250 ml of milk or yogurt or a calcium-fortified plant alternative (e.g., fortified soy drink or soy yogurt) or 50–75 g of soft cheese or 27–42 g of hard/semi-hard cheese. A range between 0 and 500 g is possible	Prefer low-fat, un-sweetened, un-salted and fermented; cheese should be modest vs milk/yogurt;		0–500
SI nat. repr. intake	Adults milk intake (g/day) was 80.0 in M and 83.8 in F Dairy products (yogurt, cheese, milk cream): 107.3 in M and 127.9 in F. Cheese: 34.7 in M and 32.0 in F In elderly milk intake was 59.9 in M and 76.3 in F, dairy products 91.2 in M and 129.5 in F, cheese 27.4 in M and 24.7 in F	more than half of milk equivalents are consumed as cheese	Milk equivalents were calculated using the formula: milk + (dairy—cheese) + (cheese * 5)	Adults: ♂ 326.1, ♀ 339.7 Elderly: ♂ 260.7, ♀ 304.6
EAT–Lancet 2.0	Milk or equivalents (e.g., cheese): 250 g/day (0–500 g/day)			≈250 (0–500)
EAT–Lancet	Milk or equivalents (eg, cheese): 250 g/day (0–500 g/day)			≈250 (0–500)
NNR 2023	Intake of between 350 to 500 ml low fat milk and dairy products per day is sufficient to meet dietary requirements of calcium, iodine and vitamin B12 if combined with adequate intake of legumes, dark green vegetables and fish (varies among different species)	If consumption of milk and dairy is lower than 350 gram/day, products may be replaced with fortified plant-based alternatives or other foods		350–500
DK	About 250 ml milk or dairy products a day is adequate About 20 g of cheese (1 slice) a day is adequate	Prefer fermented, low-fat/unsweetened; limit high salt cheeses; Choose mainly skimmed milk or buttermilk	Unclear if cheese is included in the 250 ml/day of dairy or recommended in addition; we assume it is included	250
FI	350 to 500 g/day of fat-free or low-fat dairy products is sufficient to satisfy the requirement	Plant-based beverages fortified with Ca, vit. D, iodine and vit. B12 should be preferred as alternatives to milk		350–500
SE	Eat dairy products every day			NA
NO	It is recommended to eat or drink 3 servings of milk or dairy products each day 1 serving could be: glass of (soured) milk (1.5 to 2 dl), small cup of yogurt (125 to 150 g), two slices of cheese (20 g)	Two of the servings should be milk, fermented milk or yogurt; plant-based drinks can provide many of the same nutrients		450–600
DE	Have some milk and dairy products daily	Unsweetened. Plant-based drinks can be an alternative to milk and dairy products		NA
AT	Consume 2 portions of milk and dairy products daily (3 portions for vegetarians); ideally, 2 portions of 'white' dairy and 1 portion of 'yellow' dairy (i.e., cheese). One portion corresponds to a glass or cup (150–200 ml) or 2 thin, palm-sized slices of cheese	Prefer lower-fat and unsweetened products. Those who consume less milk and yogurt should opt for unsweetened plant-based products (e.g., soy drinks) with added calcium, vitamin B12, and vitamin B2	Portion multiplied by 2 or by 3	≈300–400 *(omnivorous)* ≈450–600 *(vegetarian)*
ES	It is recommended to consume a maximum of 3 servings per day, with no added sugars and low salt. Serving: 200–250 mL milk, 85–125 g fresh cheese, 40–60 g cured cheese, 125 g yoghurt	Due to the high environmental impact of dairy products, it is suggested to reduce the number of daily servings if other animal-source foods are consumed	Assuming 1 serving = 200–250 g milk equivalents → max 3 servings/day = ≤ 600–750 g/day	≤ 600–750
FR	Dairy products (milk, yoghurt, cheese, cottage cheese): 2 per day	Prefer plain/unsweetened; moderate cheese; consider fortified alternatives if dairy excluded	Serving = 200–250 g milk equivalents	≈400–500
CN	A wide variety of dairy products should be consumed; a daily intake equivalent to 300 g of liquid milk is recommended			300
US	3 cup-equivalents of dairy per day	Low-fat/fat-free dairy encouraged; fortified soy beverage can count as dairy equivalent	1 cup-equivalent ≈ 240 g milk → 3 cups ≈720 g/day	≈720
UK	Some dairy or dairy alternatives	Choose lower-fat and lower-sugar		NA
AU	At least 2½ serves/day of reduced fat milk, yoghurt, cheese or alternatives. Serve is 200–250 ml or equivalent	Prefer mostly reduced fat; unsweetened; fortified alternatives acceptable	1 serve ≈240 g milk eq → 2.5 serves ≈ 600 g/day	> 600

In last column (standardised intake recommendation) dairy quantities are expressed as milk equivalents; serving‑based guidance converted to grams using the portion sizes specified in each guideline or assumed

**Table 10:** Quantitative and qualitative recommendations for meat (excluding fish) across dietary guidelines, expressed as g/day (as consumed)

Guideline	Verbatim quantitative recommendation (English translation)	Qualitative/extra notes (non-quantitative)	Assumptions/conversions	Standardised food group intake recommendation (total meat as consumed g/day; fish excluded)
SNG2025	The diet can include around 43 g of meat per day (range 0*–*86); on average up to a maximum of 300 g of meat or three portions per week	Prefer white, unprocessed meat (chicken, turkey). Red and processed meats “as rarely as possible”	Weekly cap 300 g/week → 42.9 g/day (rounded ≈43)	0–43
SI nat. repr. intake	Meat and meat products			Adults: ♂ 208.6, ♀ 140.8; Elderly: ♂ 188.7, ♀ 141.1
EAT–Lancet 2.0	Beef, pork, or lamb: 15 g/day (0–30 g/day) Chicken and oth. poultry: 30 g/day (0–60 g/day)		It is not clear if there is AND or OR connection between different types of meat	red: 15 (0–30) white: 30 (0–60)
EAT–Lancet	Beef and lamb: 7 g/day (0–14 g/day) Pork: 7 g/day (0–14 g/day) Chicken and other poultry: 29 g/day (0–58 g/day)	Emphasis on low red meat; fish separate table	Total meat = 7 + 7 + 29 = 43 g/day	≈43
NNR 2023	Consumption of red meat should be low and not exceed 350 g/week ready-to-eat (cooked) weight. For environmental reasons the consumption of red meat should be considerably lower than 350 g/week	Processed red meat should be as low as possible	350 g/week → 50 g/day (applies to red meat only)	Total meat: NA; red meat ≤ 50
DK	About 350 g of meat a week is adequate	Limit beef and lamb; processed meat as little as possible	350 g/week → 50 g/day	≈50
FI	Maximum about 350 g of red meat per week Reducing consumption of poultry from the current amount is recommended	Encourage lower intake; processed meats minimized. Reduction of red meat consumption should not be replaced by poultry meat	350 g/week → 50 g/day (red meat only). Total meat not explicitly defined as one number	Total meat: NA; red meat ≤ 50
SE	Eat no more than 350 g of red meat a week, cooked			Total meat: NA; red meat ≤ 50
NO	The amount of red meat should be limited to 350 g per week or less	Limit processed meat; prefer leaner/white options if eating meat		Total meat: NA; red meat ≤ 50
DE	If you eat meat and sausage, do not consume more than 300 g a week	Those who eat little or no animal products must ensure B12 supplementation	300 g/week ÷ 7 = 42.9 g/day (upper limit)	≤ 43
AT	If you eat meat, limit your meat consumption to 1 portion of lean meat or lean sausage per week. Alternatively, you can eat an additional portion of fish or meat per week	Eat red meat (e.g., beef, pork, lamb) and processed meats less frequently	Assumption a portion is 140 g 140/7 = 20	≤ 20
ES	It is recommended to consume from 0 up to a maximum of 3 servings of meat per week… Each serving equals 100–125 g	Prioritise white meat (poultry, rabbit); minimise processed meat	Max: 3 × (100–125)/7 = 42.9–53.6 g/day → reported as ≤ 53.6 (upper bound)	≤ 43–53.6
FR	Prefer poultry and limit other meat (pork, beef, veal, sheep/lamb and offal) to 500 g/week… Limit processed meats to 150 g/week	Prefer poultry; limit red meat and processed meats due to health risks	500/7 = 71.4 g/day (red meat category); 150/7 = 21.4 g/day (processed). Poultry amount not specified → total cannot be summed	Total meat: NA; red meat ≤ 71.4; processed ≤ 21.4
CN	The recommended weekly intakes: poultry and meat 300–500 g	Fish is a better choice, smoked and cured meat should be avoided	300 g/week ÷ 7 = 43—71 g/day	43–71
US	Meats, Poultry, Eggs (ounce eq/wk): 26	Typically: choose lean; limit saturated fat; processed meats are not encouraged	1 oz-eq = 28.35 g, 26 × 28.35 /7 = 105 g/day (including eggs), assuming 50 g egg/day → meat 55 g/day	≈55
UK	If you eat more than 90 g of red or processed meat per day, try to cut down to no more than 70 g/day	Lean cuts, choose low saturated fat		NA (total); red + processed ≤ 70
AU	Limit red meat to a maximum of 455 g cooked lean red meat per week	Advice also typically discourages processed meats; poultry/lean choices preferred	455/7 = 65.0 g/day (red meat cap). Total meat incl. poultry not specified as one number	Not quantified (total); red meat ≤ 65/day (avg)

**Table 11:** Quantitative and qualitative recommendations for eggs across dietary guidelines expressed as g/day (as consumed)

Guideline	Verbatim quantitative recommendation (English translation)	Qualitative / extra notes (non-quantitative)	Assumptions / conversions	Standardised food group intake recommendation (g/day, edible egg mass)
SNG2025	The diet may include up to three eggs per week (0–175 g/week or 0–25 g/day). One portion corresponds to approximately one egg (50 g edible part).”	Eggs included from mixed dishes. Prefer cooked, if fried → with minimal oil	Value explicitly given in guideline (25 g/day); alternative calculation: 3 × 50 g / 7 = 21.4 g/day	≤ 25 (or ≤ 21.4 if using 3 × 50 g/week)
SI nat.repr. intake	“Fresh and food-incorporated eggs… Adults: 43.5 g/day (men), 36.0 g/day (women); Elderly: 41.1 g/day (men), 29.3 g/day (women).”	Observational intake; includes eggs consumed as such and in composite dishes		Adults: ♂ 43.5, ♀ 36.0 Elderly: ♂ 41.1, ♀ 29.3
EAT–Lancet 2.0	Eggs: 15 g/day (0–25 g/day)			≈15 (0–25)
EAT–Lancet	Eggs: 13 g/day			≈13 (0–25)
NNR 2023	A moderate intake of egg may be part the diet			NA
DK	A couple of times a week		“Couple of times” assumed 3 times a week, 1 egg = 50 g edible Portion size assumed 50 g edible; 3 × 50 / 7 = 21.4 g/day	≈21.4
FI	Up to one egg per day including eggs used in cooking and baking	Stricter advice for people with high LDL cholesterol, diabetes or cardiovascular disease	Portion size not specified → assumed 1 egg = 50 g edible	≤ 50
SE	We don't have any specific advice about eggs or poultry			NA
NO	Eggs can be part of a healthy and varied diet			NA
DE	NA	Eggs are grouped together with meat and fish		NA
AT	With meat and fish: up to 3 eggs/week; without meat and fish: up to 4 eggs per week		Assumed 1 egg = 50 g edible; 3–4 eggs/week → 21.4–28.6 g/day	≤ 21.4 (omnivorous) / ≤ 28.6 (vegetarian)
ES	Up to four medium eggs per week	Consume with healthy foods, such as salads, vegetables, fish; avoid combinations with foods rich in saturated fats	Medium egg defined as 53–63 g edible; (4 × 53–63) / 7 = 30–36 g/day	≤ 30–36
FR	NA			NA
CN	The recommended weekly intakes: eggs 300-350 g		divided by 7	43–50
US	Meats, Poultry, Eggs (ounce eq/wk): 26		1 oz-eq = 28.35 g, 26 × 28.35 / 7 = 105 g/day (including eggs), assuming 55 g meat/day → egg 50 g/day	≈50
UK	Choose beans and pulses, fish, eggs, meat and other proteins	Eggs grouped with other protein foods; no numeric limit		NA
AU	Eggs can be eaten as an alternative to meat; up to 2 eggs per week		Assumed 1 egg = 50 g edible; 1–2/week → 7–14 g/day	≤ 14

**Table 12:** Quantitative and qualitative recommendations for fats & oils across dietary guidelines, expressed as g/day (as consumed)

Guideline	Verbatim quantitative recommendation (English translation)	Qualitative / extra notes (non-quantitative)	Assumptions / conversions	Standardised food group intake recommendation (g/day, as consumed)
SNG2025	Recommended daily intake is approximately 25 g of fats, obtained from basic foods (e.g., avocado, olives, fatty fish) and/or from plant oils (olive, sunflower, rapeseed, corn, pumpkin seed, flaxseed, walnut)	Prefer plant oils; fats/oils not strictly necessary if fat comes from whole foods; avoid coconut & palm fat, butter, lard, ghee (high in saturated fat)	assuming that 25 g is fats from basic food + oils → the amount of oil should be smaller than 25 g	≤ 25
SI nat. repr. intake	Reported as “Fats and oils” intake			Adults: ♂ 28.2; ♀ 22.6; Elderly: ♂ 26.8; ♀ 26.4
EAT–Lancet 2.0	Unsaturated plant oils: 40 g/day (20–80 g/day) Palm and coconut oil: 6 g/day (0–8 g/day) Lard, tallow, and butter: 5 g/day (0–10 g/day) included with dairy and meats		Not clear if there is AND or OR connection between different types of fats	Total amount: NA
EAT–Lancet	Palm oil: 6.8 g/day (0–6.8 g/day) Unsaturated oils: 40 g/day (20–80 g/day) Lard or tallow: 5 g/day (0–5 g/day)		Not clear if there is AND or OR connection between different types of fats	Total amount: NA
NNR 2023	It is recommended to consume at a minimum of 25 g/day vegetable oil (or similar amounts of fatty acids from whole foods)	limiting the consumption of butter and tropical oils		≥ 25
DK		choose vegetable oils (rapeseed and olive oil) rather than solid fats, (butter and coconut oil). Limit butter, choose hummus or a little pesto instead		NA
FI	A minimum of 25 g of vegetable oils should be used daily	The use of butter and tropical oils should be limited		≥ 25
SE		Choose rapeseed oil and other Nyckelhåll-labeled fats in cooking and on sandwiches		NA
NO		Choose vegetable oils and soft margarines rather than butter; limit saturated fat		NA
DE	NA	Plant oils preferred; watch “hidden fats”		NA
AT	Oils, fats, nuts & seeds — daily: 2 portions. A portion oil ≈1 tbsp.	Use butter/margarine/lard and high-fat dairy products sparingly. prefer low-fat cooking methods	Assumed 1 tbsp oil ≈10 g, assume both 2 portions as oil ≈20 g/day maximum (since portions may be nuts/seeds instead)	≤ 20
ES	Use olive oil in all meals, as a dressing and in the preparation of food. One serving is 10 ml	Reduce saturated fats (butter and other animal fats)	Assumed 3 meals per day and g = mL: 3 × 10 = 30 g/day	30
FR	Added fats—oil, butter and margarine—can be consumed every day in small quantities	Favour rapeseed, nut and olive oil		NA
CN	The daily intake of cooking oil for an adult should be less than 25–30 g/day			< 30
US	Oils (grams/day) 27 g/day			≈27
UK	Choose unsaturated oils and spreads, and eat in small amounts			NA
AU	Replace foods high in saturated fat (butter, cream, coconut/palm oil) with foods high in polyunsaturated/monounsaturated fats (oils, spreads, nut pastes, avocado)			NA

A summary of all standardised values is provided in Supplementary Table S1.

Overall, the **SNG2025** show a high degree of alignment with contemporary international and national dietary guidelines, with most quantitative recommendations falling well within ranges already adopted elsewhere. Across food groups, Slovenian targets are generally comparable to those used in other compared guidelines and international reference diets, rather than representing outliers.

For grains and cereals (Table 2), the Slovenian recommendation (≥ 230 g/day, dry weight) is closely aligned with the quantitative ranges implied in the EAT–Lancet reference diets and is comparable to recommendations in Spain and Austria, which also emphasise substantial cereal intake with a defined whole-grain component. While Nordic guidelines specify minimum whole-grain targets rather than total cereal mass, the underlying dietary pattern is broadly consistent.

For potatoes and other starchy tubers (Table 3), Slovenia’s recommendation (≈200 g/day) corresponds closely to guidance in Spain and Austria, where potatoes remain an established component of traditional diets. Although EAT–Lancet reference diets recommend lower amounts (≈50 g/day), several European national guidelines similarly treat potatoes as a regular starchy food rather than a marginal item, placing Slovenia well within common European practice.

For legumes (Table 4), Slovenian recommendations (≥ 75 g/day, dry weight) closely match the EAT–Lancet target and are comparable to the upper range of recommendations in Spain and Austria, where legumes are explicitly promoted as core protein-rich foods. While some guidelines express this advice qualitatively, Slovenia’s approach differs mainly in its explicit quantification, not in direction or intent.

For fruit (Table 5) and vegetables (Table 6), Slovenia aligns strongly with international consensus. The Slovenian lower-bound targets (≥ 200 g/day fruit; ≥ 300 g/day vegetables) fall within the range of most compared guidelines and are identical or very close to several of them**.** These food groups show the greatest convergence across most guidelines, although some countries recommend somewhat higher minimum intakes for vegetables; for example, Spain recommends ≥ 450–600 g/day. Slovenian values do not materially differ from prevailing European norms.

For nuts and seeds (Table 7), the Slovenian recommendation (≥ 30 g/day) closely mirrors the EAT–Lancet reference intake and is consistent with many guidelines. Differences across guidelines in this group largely reflect variation in whether intakes are expressed daily or weekly, rather than substantive disagreement.

For fish and seafood (Table 8), the Slovenian target (≈29 g/day, derived from weekly advice) closely matches the EAT–Lancet optimum (≈28 g/day) and overlaps with ranges recommended in Austria and Germany. Notably, some guidelines recommend lower amounts than Slovenia, such as Austria (≈21 g/day), while others, particularly Mediterranean countries such as Spain (≥ 53 g/day), specify higher minimum intakes.

For milk and dairy products (Table 9), Slovenia’s recommended range (0–500 g/day milk equivalents) overlaps with nearly all European guidelines. Several countries recommend lower quantitative intakes than the Slovenian upper range, including Denmark (≈250 g/day), China (≈300 g/day), and Austria (≈300–400 g/day), while international reference diets such as EAT–Lancet similarly operate within a broad 0–500 g/day framework. Thus, Slovenian guidance does not exceed commonly used international upper bounds. It should also be noted that several guidelines (e.g., Spain, Denmark, the United Kingdom, and the United States) explicitly include fortified plant-based alternatives within recommended dairy intakes, allowing the advised amounts to be met entirely through non-dairy products.

For total meat consumption (Table 10), Slovenia’s upper limit (≤ 43 g/day) is closely aligned with EAT–Lancet and German recommendations. However, Austria recommends even lower total meat intake (≤ 20 g/day). In several guidelines the limits are set specifically for red and processed meat intake, but not for total meat intake.

For eggs (Table 11), Slovenia’s limit (≤ 25 g/day) lies above several international recommendations, including EAT–Lancet (≈13–15 g/day), Denmark (≈21 g/day), Austria (≤ 21 g/day), and Australia (≤ 14 g/day).

Finally, for fats and oils (Table 12), Slovenia provides explicit food-based quantitative guidance (≤ 25 g/day), whereas many other guidelines treat fats primarily as nutrients rather than foods. Despite this difference in framing, the Slovenian limit is fully compatible with the qualitative emphasis on limiting added fats found across most international and national guidelines.

Comparison with observed Slovenian intake (Si.Menu 2017/18) revealed notable discrepancies across several food groups. Fruit intake is below the SNG2025 minimum of 200 g/day in adult men (162.2 g/day) but meets or exceeds this threshold in adult women and in the elderly (Table 5). Vegetable intake is consistently much lower than the recommended ≥ 300 g/day in all groups, with observed values of only 158.3–163.2 g/day, i.e., approximately 46–47% below the target (Table 6). The largest shortfalls are observed for legumes and nuts and seeds: legume intake reaches only 5.8–10.7 g/day versus the recommended ≥ 75 g/day, corresponding to a deficit of about 86–92% (Table 4), while nuts and seeds are consumed at only 4.5–9.5 g/day compared with the recommended ≥ 30 g/day, i.e. about 68–85% below the target (Table 7). Fish and seafood intake is also generally below the Slovenian target of ≈29 g/day, with observed intakes of 18.2–26.5 g/day (Table 8). By contrast, milk and dairy intake falls within the broad Slovenian recommended range of 0–500 g/day in all groups (Table 9). Meat intake substantially exceeds the Slovenian upper limit of ≤ 43 g/day in all groups, reaching 140.8–208.6 g/day in adults and 141.1–188.7 g/day in the elderly, i.e., approximately 227–385% above the limit in adults and 229–339% above the limit in elderly (Table 10). Egg intake is close to the Slovenian upper limit of ≤ 25 g/day but exceeds it in adult men and in both elderly groups (Table 11). This should nevertheless not be interpreted as adequate compliance, because the SI.Menu values represent mean population intake, whereas the SNG2025 value for eggs is a maximum recommended intake; thus, even when the mean is close to the limit, roughly half of the population can be expected to consume more than recommended. Fats and oils are also slightly above the recommended limit of ≤ 25 g/day in all groups, with observed intakes of 26.4–28.2 g/day, i.e., about 6–13% above the limit (Table 12). For grains/cereals, total observed intake is broadly in line with or above the Slovenian minimum recommendation of ≥ 230 g/day (Table 2), whereas potato intake remains below the recommended ≈200 g/day in all groups, corresponding to a shortfall of about 50–62% (Table 3).

## Discussion

### Principal findings

This study demonstrates that the SNG2025 are highly aligned with major international dietary frameworks, including the EAT–Lancet reference diet and comparable European guidelines. The highest degree of convergence was observed for fruit and vegetables, where recommended intake levels (≥ 200 g/day for fruit; ≥ 300 g/day for vegetables) are broadly consistent across international guidelines. In contrast, greater variability is observed across protein-rich food groups, particularly for meat, fish, dairy, and eggs, reflecting differences in both the magnitude and framing of recommendations [3, 11]. A defining feature of the SNG2025 is the systematic use of quantitative, food-based thresholds across all major food groups, enabling direct comparability and supporting translation into policy and practice.

When formulating FBDGs, a central and persistent challenge is how to address populations with highly heterogeneous baseline dietary patterns, while simultaneously keeping recommendations understandable and actionable for the general public. Guideline developers must balance scientific precision with communicative simplicity, a tension that is evident across many contemporary national and international recommendations. According to FAO, FBDGs are intended to provide a basis for public food and nutrition, health and agricultural policies and for nutrition education programmes, and to provide advice on foods, food groups and dietary patterns to the general public [32].

A common strategy adopted by several countries is the use of qualitative, directional messages such as “eat more” or “eat less.” For example, the UK Eatwell Guide recommends “eat less red and processed meat” and “eat more beans and pulses.” While such statements are intuitively accessible, their practical interpretation depends strongly on the individual’s habitual diet. For a person who already consumes red meat only once per week, the instruction to “eat less” provides little actionable guidance (e.g., less than current intake, less than population average, or less than undefined threshold). Conversely, advising someone who already consumes legumes multiple times per day to “eat more beans and pulses” may be equally inappropriate. In this sense, purely qualitative guidance risks being either redundant or misleading when applied across diverse dietary contexts.

Some guidelines partially address this limitation by complementing qualitative advice with numerical frequency-based recommendations. For instance, the UK guidance specifies “two portions of sustainably sourced fish per week, one of which should be oily,” and “at least five portions of a variety of fruit and vegetables per day.” Although portion sizes are not always explicitly defined in grams, the use of numerical frequencies provides a clearer reference point than unqualified statements.

The German FBDG, for example, place strong emphasis on the overall dietary pattern as illustrated by the DGE Nutrition Circle, which communicates recommended proportions of major food groups. This graphical representation was derived from diet optimisation modelling. The optimisation results were also used to inform the public-facing messages that form part of the German FBDG; however, the messages themselves intentionally contain as few explicit quantitative recommendations as possible. Quantitative recommendation (g or ml per day of week) is provided only for meat and beverages. Semi-quantitative (portions per week or per day) are provided for fish, nuts, fruits and vegetables, while other food groups are communicated primarily in qualitative or proportional terms.

Furthermore, the Canadian FBDG contain no explicit quantitative intake recommendations for food groups at all, relying entirely on qualitative guidance and visual representation of a healthy plate. This approach allows policymakers and nutrition professionals to draw on numerical values from the underlying scientific evidence, while enabling individuals and the general public to apply the guidelines flexibly, according to personal preferences and dietary habits.

By contrast, the Spanish FBDG provide quantitative advice for most food groups, typically expressed both in grams and in portions, thereby offering more explicit numerical guidance alongside qualitative recommendations.

Reflecting these challenges, SNG2025use a combined approach: quantitative recommendations expressed as grams per day or per week, often accompanied by explanations in terms of portions or servings. This approach improves transparency and facilitates comparison across guidelines and with observed dietary intake data. However, even fully quantitative recommendations typically define intake ranges (e.g., meat intake from zero to 40 g/day, legumes at ≥ 50 g/day), acknowledging dietary flexibility and cultural diversity. It is mathematically impossible to guarantee that all combinations of food intakes within such ranges will meet all nutrient requirements under every scenario.

Despite these inherent limitations, we argue that guidelines expressed using explicit quantitative values represent a methodological improvement over purely qualitative advice. Numeric targets provide clearer benchmarks for monitoring population intake, enable meaningful comparisons between countries and dietary models, and support more precise alignment between recommendations and nutritional adequacy assessments. When combined with qualitative guidance on food quality and dietary patterns, quantitative recommendations offer a more robust and transparent foundation for contemporary food-based dietary guidelines.

An additional advantage of quantitative dietary recommendations expressed as explicit amounts or defined intake ranges is that they enable modelling of both environmental and health impacts using established assessment frameworks. Such analyses are not feasible when guidance is limited to purely qualitative statements, such as generic “eat less meat” advice, which lacks a defined reference point for calculation.

### Convergence and divergence across food group recommendations

A clear pattern emerging from the comparative analysis is that dietary guidelines show markedly different degrees of convergence across food groups. For some categories, most notably vegetables and fruits, recommendations are strikingly consistent across countries and guideline frameworks. Despite differences in phrasing, operationalisation, and portion definitions, most contemporary guidelines converge on daily intakes in the range of approximately 400–800 g of fruits and vegetables combined, with vegetables generally accounting for the larger share. This convergence likely reflects a robust and mature evidence base linking high fruit and vegetable intake to reduced risk of non-communicable diseases, as well as broad agreement on their favourable nutrient density and low environmental impact per unit of weight.

In contrast, substantial divergence is observed for other food groups, particularly fish and seafood. Even among recent, sustainability-aware guidelines, recommended intakes differ widely in both absolute quantities and framing (minimum intake, target range, or frequency-based advice). For example, Austrian dietary guidelines translate to an intake of approximately 21 g/day of fish and seafood as an indicative target, whereas Spanish guidelines frame fish consumption as a minimum recommendation, corresponding to values of 53 g/day or more. Similar variability is observed across other guidelines: EAT–Lancet recommends about 28–30 g/day, Germany about 21.4–42.8 g/day, Nordic-related guidelines generally about 43–64 g/day, Denmark about 50 g/day, the United Kingdom at least 40 g/day, and China about 43–71 g/day. This illustrates substantial heterogeneity not only in the recommended quantities, but also in whether the guidance is framed as a minimum intake, a target range, or an approximate reference value. Several factors likely contribute to this heterogeneity. First, the evidence base for fish intake is multifaceted, combining cardiometabolic benefits (e.g. long-chain omega-3 fatty acids) with concerns related to contaminants, overfishing, and ecosystem impact. Second, regional availability, culinary traditions, and cultural norms strongly influence how fish is positioned within national food patterns. Third, some guidelines emphasise nutrient substitution logic (fish as a replacement for red or processed meat), while others frame fish primarily as a food delivering specific nutrients, which leads to different quantitative conclusions.

Several additional findings highlight important sources of variability across dietary guidelines. First, recommendations for legumes show substantial heterogeneity, both in quantitative targets and in their positioning within the dietary pattern. While some guidelines treat legumes as a core food group, others integrate them within broader plant-based categories, limiting direct comparability. In the case of Germany, the comparatively low quantitative value should also be interpreted with caution: according to the DGE FAQ [30] comparing German and Austrian recommendations, the Austrian category explicitly includes legumes and products made from them, with tofu and tempeh forming a substantial part of that category, which makes direct comparison more difficult and may partly explain the lower German figure.

Second, differences in substitution frameworks are evident in how dairy and fortified plant-based alternatives are treated. Some guidelines explicitly promote plant-based substitutes as replacements for dairy, whereas others provide less explicit or more fragmented guidance.

Third, variability is also observed in the framing and quantification of nuts, which are inconsistently treated either as a distinct food group or as part of broader categories such as plant-based protein sources. Taken together, these findings suggest that inter-guideline agreement is highest for food groups with clear and consistent health signals (e.g., fruit and vegetables), whereas greater divergence occurs where nutritional, environmental, and cultural considerations intersect. This has important implications for cross-country comparisons and for the interpretation of alignment between national guidelines and global reference diets.

In contrast to vegetables and fruits, potatoes and other starchy tubers are rarely assigned explicit quantitative intake targets in contemporary dietary guidelines and are instead commonly embedded within broader categories of starchy or carbohydrate-rich foods. As a result, comparability across guidelines is limited, and any attempt to standardise potato intake recommendations requires the use of proxy assumptions derived from combined groups such as “starchy foods” or “starchy vegetables”. This reflects not only methodological differences but also the ambiguous nutritional positioning of potatoes, which are variably framed as vegetables, staple carbohydrate sources, or energy foods depending on cultural context and dietary tradition.

Taken together, these findings suggest that high inter-guideline agreement is more likely for food groups with strong, unambiguous health signals and relatively low contextual constraints, whereas greater divergence persists for foods where health, environmental, cultural, and food-system considerations intersect more strongly. This has important implications for cross-country comparisons, dietary modelling, and the interpretation of “alignment” between national dietary guidelines and global reference diets.

### Fats

An important methodological exception across dietary guidelines concerns fats, which are frequently not treated as a food group in the same sense as other recipe-level foods purchased and consumed as discrete items (e.g. oils, butter, lard, palm oil, coconut fat, seed oils, olive oil). Instead, fat recommendations are often formulated at the nutrient level, focusing on total fat intake or fatty-acid classes and implicitly including fats that are intrinsic to foods from other groups, such as meat, nuts, seeds, avocado, and dairy products. While this nutrient-based approach is understandable—given that fats as nutrients are essential and are recommended to contribute a substantial share of total dietary energy—it also reflects an implicit distinction between fats as nutrients and fats as foods. Fats consumed as standalone foods are highly energy-dense and can substantially increase caloric intake with limited contributions of protein, minerals, and vitamins, whereas fats embedded within whole foods typically co-deliver other essential nutrients.

### Recommendations vs. actual intake

The findings underscore a significant 'implementation gap' between the scientifically grounded SNG2025 targets and the current dietary habits of the Slovenian population. This suggests that while the Slovenian guidelines are robust and internationally comparable, their successful adoption will require substantial public health efforts. The shift towards plant-forward patterns, as emphasized in SNG2025 through higher legume and whole grain targets, represents a major departure from current Slovenian consumption trends. Therefore, the SNG2025 should be viewed not only as a nutritional benchmark but also as a strategic tool for future dietary interventions aimed at narrowing these identified gaps. At the same time, implementation should be accompanied by validated tools for monitoring adherence to the guidelines. Several countries have already developed such indices, including the Healthy Eating Index (HEI-2020) in the United States and the Norwegian Dietary Guideline Index (NDGI) [33, 34]. These indices enable population-level surveillance, evaluation of interventions, and identification of groups with particularly low adherence.

### Strengths and limitations

#### Strengths

The main strength of this study is the systematic standardisation of diverse national and international FBDGs into a standardized quantitative format (g/day, milk equivalents), enabling direct comparison, enabling direct cross-country comparability. By including influential recently updated FBDGs (e.g., EAT-Lancet, NNR 2023), the study provides a contemporary reference framework for the SNG2025. In addition, the inclusion of nationally representative intake data (Si.Menu) provides contextual insight into the gap between recommended and observed dietary patterns in the Slovenian population.

#### Limitations

This study also has several limitations. First, the selection of guidelines was structured and purposive, rather than based on a fully systematic review. Second, although recommendations were standardised into common quantitative units, the underlying energy reference frameworks of the original guidelines (e.g. 2,000 vs. 2,500 kcal/day) were not adjusted. Third, standardisation required simplification of heterogeneous food classifications, including the mapping of mixed or differently structured food groups into harmonised categories, which may have introduced some degree of imprecision. Fourth, comparison with nationally representative intake data should be interpreted with caution, because observed intake and guideline recommendations are conceptually different: intake surveys reflect mean population consumption, whereas guidelines may define minimum, maximum, or target values.

### Future research

Future research should evaluate the potential health and environmental impacts of shifting current dietary intake in Slovenia towards the SNG2025. This may include modelling projected changes in dietary risk factors and clinical markers, alongside estimates of greenhouse gas emissions, land use, and water footprints using comparative risk assessment and life cycle assessment methods. In addition, further work could monitor adherence to the guidelines and examine disparities in uptake across socioeconomic groups, regions, and institutional settings such as hospitals and workplaces.

## Conclusions

This comparative analysis demonstrates that the SNG2025 are well aligned with contemporary international and national FBDGs across all major food groups. For plant-based food groups—particularly vegetables, fruits, whole grains, legumes, and nuts—the SNG2025 closely match international consensus and reference diets such as the EAT–Lancet frameworks and Mediterranean-oriented national guidelines. For animal-source foods, including meat, eggs, fish, and dairy products, Slovenian quantitative limits fall within widely used international ranges and are, in several cases, less restrictive than those adopted by other European countries. Observed differences between guidelines largely reflect variation in framing, cultural dietary patterns, and the degree of quantitative specification rather than substantive disagreement on nutritional principles. Overall, these findings support the scientific robustness, international comparability, and policy relevance of SNG2025, while also illustrating the value of transparent quantitative standardisation for cross-country comparison and evaluation against nationally representative dietary intake data.

## Supplementary Information

Below is the link to the electronic supplementary material.

Supplementary Material 1

## Data Availability

No datasets were generated or analysed during the current study.

## Abbreviations

AGES: Austrian agency for health and food safety
AESAN: Agencia Española de Seguridad Alimentaria y Nutrición
AU: Australian dietary guidelines
CA: Canada’s dietary guidelines
CN: Dietary guidelines for Chinese residents
DE: German dietary guidelines (DGE)
DK: Danish official dietary guidelines
EAT–Lancet: EAT–lancet commission reference diets
FBDG: Food-based dietary guidelines
FI: Finnish nutrition recommendations
FR: French dietary recommendations (Santé publique France)
NNR: Nordic nutrition recommendations
NO: Norwegian dietary guidelines
SE: Swedish dietary guidelines
SI.Menu: Slovenian national dietary survey 2017/18
SNG2025: Slovenian nutrition guidelines 2025
UK: United Kingdom eatwell guide
US: Dietary guidelines for Americans
