# Supplementary material for: Quantitative comparison of food-based dietary guidelines: alignment with the Slovenian nutrition guidelines 2025 and Slovenian intake
Source: Eur J Nutr. 2026 Jun 2;65(4):148. doi: 10.1007/s00394-026-04009-4 (PMC13230274; doi:10.1007/s00394-026-04009-4)
Supplement: Supplementary file 1 — Supplementary Material 1 [file 394_2026_4009_MOESM1_ESM.docx]

**Supplementary material**

Supplementary Table S1. Summary of standardised food group intake recommendations across all included dietary guidelines and observed Slovenian dietary intake (SI.Menu 2017/18). All values are expressed as g/day; grains/cereals and legumes/pulses are given as dry weight; milk and dairy as milk calcium equivalents; all other food groups as consumed. NA = recommendation not quantified or not extractable.

| FBDG | grains / cereals | potatoes / starchy tubers | legumes / pulses | fruits | vegetables | nuts & seeds | fish & seafood | milk & dairy | meat | eggs | fats & oils |  |
| --- | --- | --- | --- | --- | --- | --- | --- | --- | --- | --- | --- | --- |
| SNG2025 | ≥230 | ≈200 | ≥75 | ≥200 | ≥300 | ≥30 | ~29  (range 0–64) | 0–500 | 0-43 | ≤25 | ≤25 |  |
| SI nat. repr. intake | Adults:  ♂ 307.2,  ♀ 239.2;  Elderly:  ♂ 274.1,  ♀ 231.3 | Adults:  ♂ 99.0,  ♀ 75.7  Elderly:  ♂ 99.0,  ♀ 87.8 | Adults:  ♂ 7.3,  ♀ 5.8,  Elderly:  ♂ 10.7,  ♀8.7 | Adults:  ♂ 162.2,  ♀ 226.0  Elderly:  ♂ 210.7,  ♀ 267.8 | Adults:  ♂ 163.0,  ♀ 158.3  Elderly:  ♂ 160.1,  ♀ 163.2 | Adults:  ♂ 7.7;  ♀ 9.5  Elderly:  ♂ 4.5;  ♀ 6.8 | Adults:  ♂ 26.5;  ♀ 18.2  Elderly:  ♂ 19.8;  ♀ 21.2 | Adults:  ♂ 326.1,  ♀ 339.7  Elderly:  ♂ 260.7,  ♀ 304.6 | Adults:  ♂ 208.6,  ♀ 140.8;  Elderly:  ♂ 188.7,  ♀ 141.1 | Adults:  ♂ 43.5,  ♀ 36.0  Elderly:  ♂ 41.1,  ♀ 29.3 | Adults:  ♂ 28.2;  ♀ 22.6;  Elderly:  ♂ 26.8;  ♀ 26.4 |  |
| EAT–Lancet 2.0 | ≈210 | ≈50 *(0–100)* | ≈75 *(0–150)* | ≈200 (100–300) | ≈300 *(200–600)* | ≈50 (0–75) | ≈ 30 (0–100) | ≈250 (0–500) | red: 15 (0–30)  white: 30 (0–60) | ≈15 (0–25) | total: NA |  |
| EAT–Lancet | 232 *(0–464)* | ≈50 *(0–100)* | 75 *(0–150)* | 200 *(100–300)* | ≈300 *(200–600)* | ≈50 | ≈ 28 (0–100) | ≈250 (0–500) | ≈43 | ≈13 (0–25) | total: NA |  |
| NNR 2023 | ≥90 | NA | NA | ≥200 *(or >320)* | ≥300 *(or* ≥*480)* | 20- 30 grams nuts  + seeds | 43–64 | 350 - 500 | Total: NA; red: ≤50 | NA | ≥25 |  |
| DK | ≥75 | ≈100 | ≈50 | ≥240 | ≥300 | ≈40-50 | 50 | 250 | ≈50 | ≈21.4 | NA |  |
| FI | ≥90 | NA | ≈25–50 | ≥250 (≥400) | ≥250 *(can be >400)* | nuts: 20 - 30  seeds: NA | 43–64 | 350 - 500 | Total: NA; red: ≤50 | ≤50 | ≥25 |  |
| SE | ≥90 | NA | NA | ≥200 | ≥300 | nuts: 20 - 30  seeds: NA | ~43–64 | NA | Total: NA; red meat ≤50 | NA | NA |  |
| NO | ≥90 | NA | NA | ≥250 (≥400) | ≥250 (≥400) | nuts: 20 - 30  seeds: NA | 43–64 | 450 - 600 | Total: NA; red: ≤50 | NA | NA |  |
| DE | NA | NA | ≥9.9 | ≥200 | ≥300 | 25 | ~21.4–42.8 | NA | ≤43 | NA | NA |  |
| AT | 0-280 | 0-800 | ≥26.8 *(omniv.)*  ≥35.7 *(veget.)* | ≈200 | ≈300 | ≤40 | ~21.4 | ≈300–400 (omniv.)  ≈450–600 (veget.) | ≤ 20 | ≤21.4 (omniv.)  ≤28.6 (veget.) | ≤20 |  |
| ES | 120 - 480 | NA | 28.6–60 | 240 to 360 | ≥450 *- 600* | 8.5 to 30 | ≥53.6 - 64.3 | ≤600–750 | ≤43-53.6 | ≤30–36 | 30 |  |
| FR | NA | 0-800 | ≥14 | ≥200 | ≥240 (or ≥300) | ≈20 | ~42.8 | ≈400–500 | Total: NA; red: ≤71.4; processed: ≤21.4 | NA | NA |  |
| CN | 200-300 | 50-100 | NA | 200-350 | 300–500 | NA | 43–71 | 300 | 43 - 71 | 43–50 | <30 |  |
| US | Total ≈170  Whole Grains ≥ 85 | ≈107-150 | ≈19 | ≈300 | ≈236 | ≈20 | ≥32 | ≈720 | ≈55 | ≈50 | ≈27 |  |
| UK | NA | NA | NA | ≥200 | ≥300 | NA | ≥40 | NA | Total: NA; red+processed ≤70 | NA | NA |  |
| AU | ≈240 | NA | NA | ≥300 | ≥375 | 21 | ~29 | >600 | Total: NA; red: ≤65/day | ≤14 | NA |  |
